# Vascular deficits contributing to skeletal fragility in type 1 diabetes

**DOI:** 10.3389/fcdhc.2023.1272804

**Published:** 2023-10-06

**Authors:** Adina E. Draghici, Bita Zahedi, J. Andrew Taylor, Mary L. Bouxsein, Elaine W. Yu

**Affiliations:** ^1^ Department of Physical Medicine and Rehabilitation, Harvard Medical School, Boston, MA, United States; ^2^ Cardiovascular Research Laboratory, Schoen Adams Research Institute at Spaulding Rehabilitation, Cambridge, MA, United States; ^3^ Endocrine Unit, Massachusetts General Hospital, Boston, MA, United States; ^4^ Center for Advanced Orthopedic Studies, Beth Israel Deaconess Medical Center, Boston, MA, United States

**Keywords:** T1D, skeletal fragility, microvascular disease, calcification, bone blood flow

## Abstract

Over 1 million Americans are currently living with T1D and improvements in diabetes management have increased the number of adults with T1D living into later decades of life. This growing population of older adults with diabetes is more susceptible to aging comorbidities, including both vascular disease and osteoporosis. Indeed, adults with T1D have a 2- to 3- fold higher risk of any fracture and up to 7-fold higher risk of hip fracture compared to those without diabetes. Recently, diabetes-related vascular deficits have emerged as potential risks factors for impaired bone blood flow and poor bone health and it has been hypothesized that there is a direct pathophysiologic link between vascular disease and skeletal outcomes in T1D. Indeed, microvascular disease (MVD), one of the most serious consequences of diabetes, has been linked to worse bone microarchitecture in older adults with T1D compared to their counterparts without MVD. The association between the presence of microvascular complications and compromised bone microarchitecture indicates the potential direct deleterious effect of vascular compromise, leading to abnormal skeletal blood flow, altered bone remodeling, and deficits in bone structure. In addition, vascular diabetic complications are characterized by increased vascular calcification, decreased arterial distensibility, and vascular remodeling with increased arterial stiffness and thickness of the vessel walls. These extensive alterations in vascular structure lead to impaired myogenic control and reduced nitric-oxide mediated vasodilation, compromising regulation of blood flow across almost all vascular beds and significantly restricting skeletal muscle blood flow seen in those with T1D. Vascular deficits in T1D may very well extend to bone, compromising skeletal blood flow control, and resulting in reduced blood flow to bone, thus negatively impacting bone health. Indeed, several animal and *ex vivo* human studies report that diabetes induces microvascular damage within bone are strongly correlated with diabetes disease severity and duration. In this review article, we will discuss the contribution of diabetes-induced vascular deficits to bone density, bone microarchitecture, and bone blood flow regulation, and review the potential contribution of vascular disease to skeletal fragility in T1D.

## Background

The incidence of type 1 diabetes (T1D) is increasing by 2-5% per year worldwide (1, 2) such that over 1 million Americans are currently living with T1D (3). Additionally, advancements in diabetes management have led to an increased number of adults with T1D living into later decades of life (3–6). Unfortunately, this growing population of older adults with diabetes is more susceptible to age-related health problems, including bone loss, osteoporosis, and increased fracture risk. Indeed, adults with T1D have a 2- to 3-fold higher risk of any fracture and up to 7-fold higher risk of hip fracture compared to those without diabetes (7–13). Moreover, those with T1D typically experience worse outcomes and increased complications following a fracture (14, 15). Several factors have been postulated to contribute to skeletal fragility in T1D, however none adequately account for the significantly higher fracture risk. For example, the reduced bone mineral density (BMD) observed in T1D predicts only a 1.4-fold increased risk of hip fracture (16), which is far below the observed 5- to 7-fold higher risk associated with T1D (17). Both diabetes-related vascular disease, as well as poor glycemic control (18), have emerged as potential risk factors for impaired bone health. Higher HbA1c values, which indicate worse glycemic control, are related to increased risk of microvascular disease (MVD) but data on the association between skeletal fragility and HbA1c are equivocal (17, 19–27). Hence, MVD itself may be the primary culprit for bone loss as opposed to glycemic control per se. Notably, most studies report worse bone microarchitecture in T1D adults with MVD (24, 28, 29). Vascular calcifications have also been associated with increased fracture risk (30–34), and thus the high prevalence of macrovascular disease in T1D may also contribute to skeletal fragility. This review article examines the impact of diabetes-induced vascular deficits on bone density and microarchitecture, and discusses the potential influence of MVD on bone blood flow regulation and resulting skeletal fragility in older adults with T1D.

## Microvascular disease, diabetes, and vascular damage

Microvascular disease (MVD) affecting small blood vessels represents one of the most serious clinical consequences of diabetes. An overwhelming majority of those with long-term T1D manifest at least one microvascular complication, with retinopathy affecting more than 70% (35), nephropathy present in about 30% (36), and neuropathy impacting up to 90% (37, 38) of older adults with T1D. Glycemic control, which declines in older adults with T1D (39), is inversely associated with risks of nephropathy and neuropathy (39, 40). Moreover, even with strict glycemic control, MVD can still develop, resulting in end-organ compromise (41–43). Although the most well-recognized end-organ targets of MVD in diabetes are the kidney, eye, and nervous system, it is likely that other tissues, such as bone, are also directly impacted. Indeed, several studies have shown greater cortical and trabecular structural deficits and lower bone strength in adults with T1D and MVD compared to their counterparts without MVD (24, 28). The association between the presence of microvascular complications and compromised bone microarchitecture indicates the potential direct deleterious effect of vascular compromise, leading to abnormal skeletal blood flow, altered bone remodeling (44), and deficits in bone structure. Several animal and *ex vivo* human studies report that diabetes induces microvascular damage within bone, including arteriole and capillary rarefaction and apoptosis (45–47), which are strongly correlated with diabetic disease severity and duration (47). In addition, vascular diabetic complications are characterized by decreased arterial distensibility and vascular remodeling with increased arterial stiffness and increased thickness of the vessel walls due to smooth muscle hyperplasia (48–50). Vascular deficits in T1D may very well extend to the bone vasculature, compromising skeletal blood flow regulation, and resulting in reduced blood flow to bone, thus negatively impacting bone health.

## Potential alterations in blood flow regulation in diabetes

Similar to other vascular beds, the circulation of bone contains an extensive network of arteries, arterioles and capillaries that provides nutrients, oxygen, and precursor cells critical for all skeletal functions (51). Maintaining skeletal integrity requires appropriate vascular supply and well-regulated blood flow to meet bone metabolic demands. Despite its critical importance, regulation of bone blood flow regulation remains poorly understood, especially in humans and in the context of disease such as diabetes. Broadly, regional regulation of blood flow results in part from a complex interplay of intrinsic local mechanisms of smooth muscle control via vascular myogenic and nitric oxide (NO)-mediated responses.

Vascular myogenic control is crucial for normal hemodynamic function and for maintaining vascular conductance, regulating tissue perfusion, and protecting downstream arterioles and capillaries from damage due to variable perfusion pressure (52–54). In response to changes in local pressure, vascular smooth muscle relaxes (i.e., vasodilation), allowing more blood flow, or contracts (i.e., vasoconstriction), thereby restricting flow. In this way, myogenic responses counter decreases in perfusion pressure with vasodilation and increases in perfusion pressure with vasoconstriction to maintain regional blood flow constant to tissue. However, alterations in vascular structure and function with aging and T1D lead to impaired myogenic vasodilatory responses (55) and heightened vasoconstrictor responses across numerous vascular beds such as muscle, skin (56–58), and retina (59). For example, older adults with T1D and MVD have impaired myogenic vasodilatory response in skeletal muscle (60), which likely contributes to the large deficit in skeletal muscle blood flow (-35%) in this population (61, 62) compared to nondiabetic controls.

Another important regulatory mechanism of blood flow is NO-mediated vasodilation. In response to increased shear stress that acts over a relatively short time (3-5 sec) (63, 64), the endothelium releases NO that dilates the vessels, allowing for increased flow, and thus playing a pivotal role in maintaining appropriate perfusion of all tissues (63–65). However, aging and T1D are associated with significant reductions in NO production and decreases in NO sensitivity, leading to blood flow reductions across numerous vascular beds. Recent work on long-term diabetes in rats indicates reduced NO-mediated vasodilation in the femoral principal nutrient artery that progresses with disease duration (46). Moreover, in humans with T1D, vasodilatory dysfunction has been identified as an early marker of microvascular complications (66–70). Indeed, structural and functional vascular alterations occur early during diabetes development, long before the manifestation of overt MVD (71–73). Vasodilatory dysfunction is present in over 35% of individuals within 5 years of T1D onset (74, 75), reducing NO-mediated vasodilation by up to 40% across almost all vascular beds (68, 76–78). NO-mediated vasodilation seems to be further impaired by aging in diabetic adults with microvascular complications (79, 80).

Despite the likelihood that the effects of diabetes and MVD on myogenic and NO vascular responses extend to bone in individuals with T1D, this important area of investigation remains largely unexplored. Given the shared pathophysiological mechanisms and the systemic nature of T1D and its associated complications, it is reasonable to hypothesize that the effects of diabetes and MVD likely also influence vascular function and blood flow regulation within the skeletal system.

## Link between vascular control, bone blood flow, and bone health

Our understanding of myogenic and NO vascular responses within the bone vasculature and their relative importance for bone health is extremely limited, particularly in the presence of diabetes and MVD. Animal data suggest that arteriolar smooth muscle in bone responds as expected to infused vasodilators and vasoconstrictors, with vasodilators increasing (81) and vasoconstrictors decreasing blood flow to bone (82–88). Moreover, animal studies suggest that NO could be one of the main mediators of blood flow to bone (81, 89–91). However, there have been very few studies investigating these mechanisms in bone in humans. In young healthy adults, our recent preliminary data have shown the presence of myogenic control and NO-mediated vasodilation in tibial bone with distinct mangnitudes and time-courses compared to skeletal muscle. In addition, in another human study of young healthy adults, blockade of endogenous NO formation reduced blood flow to femoral bone marrow as assessed by positron emission tomography (92). Although these initial findings indicate that myogenic and NO vascular responses play an important role in controlling bone blood flow, their specific role in bone blood flow control and their relationship to bone strength and structure, particularly in the presence of diabetes, remain unknown.

There are compelling reasons to suggest that altered regulation of bone blood flow has detrimental effects on skeletal health. Without adequate perfusion to supply oxygen and essential nutrients critical for bone metabolism, nearly all skeletal functions are compromised, including bone formation, maintenance, and repair. Indeed, animal studies demonstrate that regional decreases in bone blood perfusion are associated with localized declines in bone mass (93). Animal work also suggests that if vasodilation is reduced by only ~20-25%, skeletal metaphyseal and bone marrow blood flow are reduced by almost twice as much, simply due to the Poiseuille relationship between flow and vessel diameter (81). Thus, minorly compromised vasodilation can lead to marked reductions in bone blood flow, resulting in insufficient oxygen and nutrient supply for maintaining bone health. Furthermore, in longitudinal clinical studies of aging, reduced large vessel distensibility as assessed by ankle-brachial vascular index is associated with lower extremity bone loss (94). Moreover, reduced skeletal blood flow quantified as reduction in number of bone marrow blood vessels (arteries, arterioles, and capillaries) has been linked to the development of osteoporosis (95). These findings suggest that local reductions in blood flow may directly impact blood flow within bone, thus negatively impacting bone strength.

**Figure 1:**
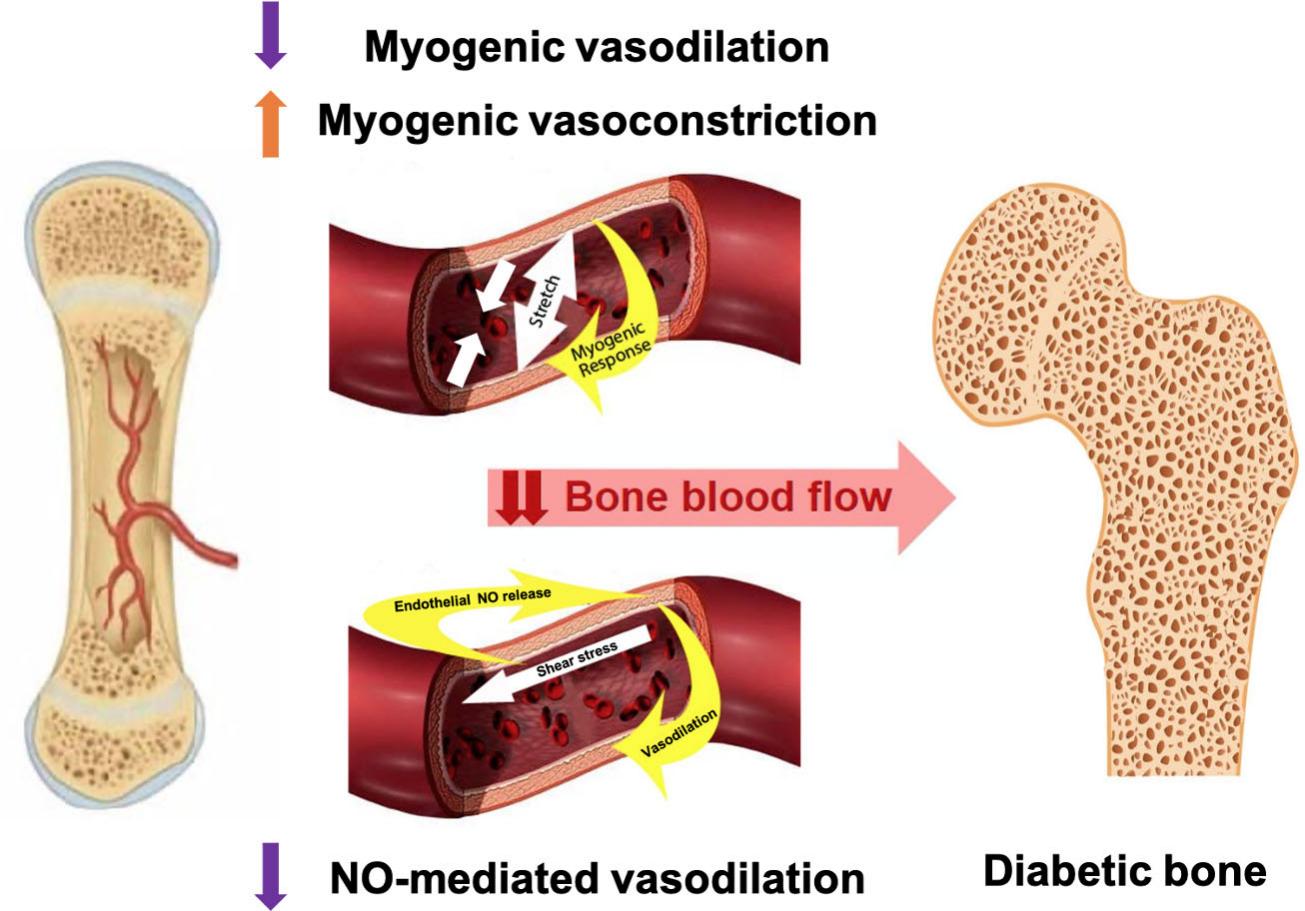
Proposed vascular alterations contributing to skeletal fragility in T1D: altered myogenic control and blunted NO-mediated vasodilation may reduce blood flow to one; contributing to bone declines in older adults with T1D.

In the context of T1D and MVD, if the bone vasculature has a diminished ability to regulate blood flow due to compromised vascular myogenic or NO-mediated mechanisms, this would lead to lesser blood flow to bone. (**Figure 1**) Consequently, this vascular impairment may contribute to skeletal fragility and increased fracture risk in adults with T1D.

## Vascular calcifications in diabetes and implications for bone blood flow

Vascular calcification, a hallmark of aging (96, 97), is accelerated in patients with diabetes (98). In fact, atherosclerotic calcification develops 10 years earlier than in those without diabetes and is often present even among asymptomatic older adults with T1D (99). The calcification of the macrovascular conduit arteries affects downstream blood flow regulation and ultimately impacts bone health. Genetically modified mice that mimic human arterial calcification show a slight increase in arterial stiffness and hyperresponsive vascular myogenic constriction (100), particularly with aging, which leads to less efficient control of local blood flow. Furthermore, several animal studies suggest that reduced aortic calcification is associated with greater NO (101–103), while accelerated calcification relates to impaired NO (104, 105). The extensive vascular dysfunction and reduced NO-mediated vasodilation in those with T1D may promote or derive from arterial calcification, leading to disrupted blood flow regulation and restricted critical flow to numerous tissues, likely to bone as well. Hence, it is not surprising that a well-established link exists between vascular disease and osteoporosis.

Several epidemiologic studies of older adults have shown an association between increased arterial calcification and bone loss particularly at the hip and spine using different imaging techniques. One study conducted with community-dwelling men over the age of 65 found that higher abdominal aortic calcification (AAC) scores assessed through lateral thoraco-lumbar radiographs were independently associated with an increased risk of non-spine fractures, particularly hip fractures (HR 1.36, 95%CI: 1.10-1.68) (33). Similarly, a case-cohort study in non-Black women aged 65 years and older found that severe AAC, evaluated from lateral spine radiographs, was associated with a higher risk of vertebral fractures (OR 2.31, 95%CI: 1.24-4.3, p<0.01) (34). Furthermore, a meta-analysis study found a significant association between coronary artery disease (CAD) and low BMD (106), while another meta-analysis demonstrated that vascular calcification was linked to lower lumbar spine and hip BMD levels, as well as an increased risk of developing osteoporosis/osteopenia (107). These studies suggest a negative association between arterial calcification and BMD as measured by dual-energy X-ray absorptiometry (DXA). Of note, DXA may artifactually overestimate BMD in the presence of vascular calcification, particularly at the lumbar spine, and thus the inverse relationship observed between arterial calcification and DXA-BMD is even more striking. These findings have been confirmed by skeletal imaging using 3D modalities such as quantitative computed tomography (QCT), which is not subject to confounding by vascular calcification. One study employing QCT found increased aortic arterial calcification was associated with decreased spine trabecular vBMD in older adults, although no association between cortical vBMD and vascular or valvular calcification was found (108). The emergence of high resolution peripheral quantitative computed tomography (HR-pQCT) allowed not only a better characterization of the tibial and radial bone microarchitecture (109, 110), but also a simultaneous assessment of lower leg arterial calcification (LLAC) in elderly individuals, those with diabetes or chronic kidney disease (111). In a study of patients with end-stage renal disease, moderate-to-severe coronary artery calcification was associated with lower tibial BMD and bone volume as assessed by HR-pQCT (112). Employing HR-pQCT to assess LLAC, a cross-sectional study in older adults found that distal tibia LLAC was correlated with lower trabecular number in male participants, and lower cortical area, lower trabecular number, and higher trabecular spacing in the female participants (113). In another study involving older participants with advanced chronic kidney disease (CKD), the presence of distal tibial LLAC was correlated with worse cortical vBMD, thickness, and porosity (114). Unfortunately, little is known about the impact of vascular calcifications on bone endpoints within diabetic populations.

Taken together, these studies highlight the complex relationship between diabetes, vascular calcification, and bone health. Diabetes is associated with vascular calcification which subsequently alters blood flow control, restricting flow to numerous vascular beds. Furthermore, LLAC is associated with worse bone health, characterized by deficits in both cortical and trabecular bone compartments across different populations, including older adults with CKD. Consequently, the presence of diabetes-associated vascular calcification within the arterial system may directly impact local bone blood flow regulation and bone microarchitecture. More research is necessary to explore the pathophysiology and clinical consequences of vascular calcifications and bone measures in the context of diabetes.

## Conclusions

Both osteoporosis and vascular disease are highly prevalent conditions that lead to profound morbidity and mortality in older adults with T1D. Although MVD affecting small blood vessels (e.g., retinopathy, nephropathy, neuropathy) has been implicated in diabetic skeletal fragility, to date, the potential contribution of bone blood vascularization to bone fragility remains poorly investigated. In this review, we discussed potential vascular mechanisms that may be present in the bone vasculature and may play a direct role in reducing blood flow supply to bone and compromising skeletal integrity in adults with T1D. The characteristics of MVD including vascular remodeling, increased arterial stiffness, as well as vascular calcification negatively impact the complex mechanisms of blood flow regulation such as myogenic and NO-mediated vascular responses. Impairments in these mechanisms have been documented in numerous other tissues in adults with diabetes. These vascular deficits likely extend to the bone vasculature and may lead to compromised blood flow supply to bone, resulting in cortical and trabecular bone deficits and increased fracture risk. Understanding the interplay between vascular disease, blood flow regulation in bone, and osteoporosis in individuals with T1D is an essential step to identify potential therapeutic interventions to improve bone health outcomes in populations with bone loss pathology.

## Author contributions

AD: Writing – original draft, Writing – review & editing. BZ: Writing – review & editing. AT: Writing – original draft, Writing – review & editing. MB: Writing – original draft, Writing – review & editing. EY: Writing – original draft, Writing – review & editing.

## References

[1] Patterson CC Dahlquist GG Gyürüs E Green A Soltész G EURODIAB Study Group. Incidence trends for childhood type 1 diabetes in Europe during 1989-2003 and predicted new cases 2005-20: a multicentre prospective registration study. Lancet (2009) 373:2027–33. doi: 10.1016/S0140-6736(09)60568-7 19481249

[2] Secrest AM Becker DJ Kelsey SF Laporte RE Orchard TJ. Cause-specific mortality trends in a large population-based cohort with long-standing childhood-onset type 1 diabetes. Diabetes (2010) 59:3216–22. doi: 10.2337/db10-0862 PMC2992785 20739685

[3] Maahs DM West NA Lawrence JM Mayer-Davis EJ. Epidemiology of type 1 diabetes. Endocrinol Metab Clin North Am (2010) 39:481–97. doi: 10.1016/j.ecl.2010.05.011 PMC2925303 20723815

[4] Dhaliwal R Weinstock RS. Management of type 1 diabetes in older adults. Diabetes Spectr (2014) 27:9–20. doi: 10.2337/diaspect.27.1.9 26246751 PMC4522896

[5] Schütt M. Multiple complications and frequent severe hypoglycaemia in ‘elderly’ and ‘old’ patients with Type 1 diabetes. Diabetes Med (2012) 29:e176–179. doi: 10.1111/j.1464-5491.2012.03681.x 22506989

[6] Miller RG Secrest AM Sharma RK Songer TJ Orchard TJ. Improvements in the life expectancy of type 1 diabetes: the Pittsburgh Epidemiology of Diabetes Complications study cohort. Diabetes (2012) 61:2987–92. doi: 10.2337/db11-1625 PMC3478551 22851572

[7] Shah VN Shah CS Snell-Bergeon JK. Type 1 diabetes and risk of fracture: meta-analysis and review of the literature. Diabetes Med (2015) 32:1134–42. doi: 10.1111/dme.12734 PMC4860902 26096918

[8] Fraser L-A Papaioannou A Adachi JD Ma J Thabane L CaMos Research Group. Fractures are increased and bisphosphonate use decreased in individuals with insulin-dependent diabetes: a 10 year cohort study. BMC Musculoskelet Disord (2014) 15:201. doi: 10.1186/1471-2474-15-201 24919660 PMC4065314

[9] Liao C-C. Increased risk of fracture and postfracture adverse events in patients with diabetes: two nationwide population-based retrospective cohort studies. Diabetes Care (2014) 37:2246–52. doi: 10.2337/dc13-2957 24804698

[10] Weber DR Haynes K Leonard MB Willi SM Denburg MR. Type 1 diabetes is associated with an increased risk of fracture across the life span: a population-based cohort study using The Health Improvement Network (THIN). Diabetes Care (2015) 38:1913–20. doi: 10.2337/dc15-0783 PMC4580610 26216874

[11] Janghorbani M Feskanich D Willett WC Hu F. Prospective study of diabetes and risk of hip fracture: the Nurses’ Health Study. Diabetes Care (2006) 29:1573–8. doi: 10.2337/dc06-0440 16801581

[12] Miao J Brismar K Nyrén O Ugarph-Morawski A Ye W. Elevated hip fracture risk in type 1 diabetic patients: a population-based cohort study in Sweden. Diabetes Care (2005) 28:2850–5. doi: 10.2337/diacare.28.12.2850 16306544

[13] Thong EP. Fracture risk in young and middle-aged adults with type 1 diabetes mellitus: A systematic review and meta-analysis. Clin Endocrinol (Oxf) (2018) 89:314–23. doi: 10.1111/cen.13761 PMC6105385 29876960

[14] Hu F Jiang C Shen J Tang P Wang Y. Preoperative predictors for mortality following hip fracture surgery: a systematic review and meta-analysis. Injury (2012) 43:676–85. doi: 10.1016/j.injury.2011.05.017 21683355

[15] Huang Y-F Shyu Y-IL Liang J Chen M-C Cheng H-S Wu C-C. Diabetes and health outcomes among older Taiwanese with hip fracture. Rejuvenation Res (2012) 15:476–82. doi: 10.1089/rej.2011.1308 22998328

[16] Maddaloni E. Bone health in subjects with type 1 diabetes for more than 50 years. Acta Diabetol (2017) 54:479–88. doi: 10.1007/s00592-017-0973-2 PMC5406751 28236093

[17] Vestergaard P. Discrepancies in bone mineral density and fracture risk in patients with type 1 and type 2 diabetes–a meta-analysis. Osteoporos Int (2007) 18:427–44. doi: 10.1007/s00198-006-0253-4 17068657

[18] Leanza G. Risk factors for fragility fractures in type 1 diabetes. Bone (2019) 125:194–9. doi: 10.1016/j.bone.2019.04.017 31059862

[19] Farr JN Drake MT Amin S Melton LJ McCready LK Khosla S. In vivo assessment of bone quality in postmenopausal women with type 2 diabetes. J Bone Miner Res (2014) 29:787–95. doi: 10.1002/jbmr.2106 PMC3961509 24123088

[20] Olmos JM Pérez-Castrillón JL García MT Garrido JC Amado JA González-Macías J. Bone densitometry and biochemical bone remodeling markers in type 1 diabetes mellitus. Bone Miner (1994) 26:1–8. doi: 10.1016/S0169-6009(08)80157-2 7950501

[21] Valerio G del Puente A Esposito del-Puente A Buono P Mozzillo E Franzese A. The lumbar bone mineral density is affected by long-term poor metabolic control in adolescents with type 1 diabetes mellitus. Horm Res (2002) 58:266–72. doi: 10.1159/000066441 12446989

[22] Heap J Murray MA Miller SC Jalili T Moyer-Mileur LJ. Alterations in bone characteristics associated with glycemic control in adolescents with type 1 diabetes mellitus. J Pediatr (2004) 144:56–62. doi: 10.1016/j.jpeds.2003.10.066 14722519

[23] Neumann T. Glycaemic control is positively associated with prevalent fractures but not with bone mineral density in patients with Type 1 diabetes. Diabetes Med (2011) 28:872–5. doi: 10.1111/j.1464-5491.2011.03286.x 21395677

[24] Shanbhogue VV. Bone geometry, volumetric density, microarchitecture, and estimated bone strength assessed by HR-pQCT in adult patients with type 1 diabetes mellitus. J Bone Miner Res (2015) 30:2188–99. doi: 10.1002/jbmr.2573 26096924

[25] Furst JR. Advanced glycation endproducts and bone material strength in type 2 diabetes. J Clin Endocrinol Metab (2016) 101:2502–10. doi: 10.1210/jc.2016-1437 PMC4891790 27115060

[26] Schneider ALC Williams EK Brancati FL Blecker S Coresh J Selvin E. Diabetes and risk of fracture-related hospitalization: the Atherosclerosis Risk in Communities Study. Diabetes Care (2013) 36:1153–8. doi: 10.2337/dc12-1168 PMC3631877 23248194

[27] Li C-I. Glycated hemoglobin level and risk of hip fracture in older people with type 2 diabetes: A competing risk analysis of Taiwan diabetes cohort study. J Bone Miner Res (2015) 30:1338–46. doi: 10.1002/jbmr.2462 25598134

[28] Abdalrahaman N. Deficits in trabecular bone microarchitecture in young women with type 1 diabetes mellitus. J Bone Miner Res (2015) 30:1386–93. doi: 10.1002/jbmr.2465 25627460

[29] Shanbhogue VV Hansen S Frost M Brixen K Hermann AP. Bone disease in diabetes: another manifestation of microvascular disease? Lancet Diabetes Endocrinol (2017) 5:827–38. doi: 10.1016/S2213-8587(17)30134-1 28546096

[30] Hirose K. Increased pulse wave velocity associated with reduced calcaneal quantitative osteo-sono index: possible relationship between atherosclerosis and osteopenia. J Clin Endocrinol Metab (2003) 88:2573–8. doi: 10.1210/jc.2002-021511 12788857

[31] Hyder JA Allison MA Criqui MH Wright CM. Association between systemic calcified atherosclerosis and bone density. Calcif Tissue Int (2007) 80:301–6. doi: 10.1007/s00223-007-9004-6 17505774

[32] Kiel DP Kauppila LI Cupples LA Hannan MT O’Donnell CJ Wilson PW. Bone loss and the progression of abdominal aortic calcification over a 25 year period: the Framingham Heart Study. Calcif Tissue Int (2001) 68:271–6. doi: 10.1007/BF02390833 11683533

[33] Szulc P. High hip fracture risk in men with severe aortic calcification: MrOS study. J Bone Miner Res (2014) 29:968–75. doi: 10.1002/jbmr.2085 PMC3935989 23983224

[34] Szulc P Samelson EJ Sornay-Rendu E Chapurlat R Kiel DP. Severity of aortic calcification is positively associated with vertebral fracture in older men–a densitometry study in the STRAMBO cohort. Osteoporos Int (2013) 24:1177–84. doi: 10.1007/s00198-012-2101-z PMC3656471 22872071

[35] Roy MS Klein R O’Colmain BJ Klein BEK Moss SE Kempen JH. The prevalence of diabetic retinopathy among adult type 1 diabetic persons in the United States. Arch Ophthalmol (2004) 122:546–51. doi: 10.1001/archopht.122.4.546 15078673

[36] Gheith O Farouk N Nampoory N Halim MA Al-Otaibi T. Diabetic kidney disease: world wide difference of prevalence and risk factors. J Nephropharmacol (2016) 5:49–56. 28197499 PMC5297507

[37] Agashe S Petak S. Cardiac autonomic neuropathy in diabetes mellitus. Methodist Debakey Cardiovasc J (2018) 14:251–6. doi: 10.14797/mdcj-14-4-251 PMC6369622 30788010

[38] Dimitropoulos G Tahrani AA Stevens MJ. Cardiac autonomic neuropathy in patients with diabetes mellitus. World J Diabetes (2014) 5:17–39. doi: 10.4239/wjd.v5.i1.17 24567799 PMC3932425

[39] Pettus JH. Incidences of severe hypoglycemia and diabetic ketoacidosis and prevalence of microvascular complications stratified by age and glycemic control in U.S. Adult patients with type 1 diabetes: A real-world study. Diabetes Care (2019) 42:2220–7. doi: 10.2337/dc19-0830 31548241

[40] Nathan DM. & DCCT/EDIC Research Group. The diabetes control and complications trial/epidemiology of diabetes interventions and complications study at 30 years: overview. Diabetes Care (2014) 37:9–16. doi: 10.2337/dc13-2112 24356592 PMC3867999

[41] Bending JJ Viberti GC Watkins PJ Keen H. Intermittent clinical proteinuria and renal function in diabetes: evolution and the effect of glycaemic control. Br Med J (Clin Res Ed) (1986) 292:83–6. doi: 10.1136/bmj.292.6513.83 PMC1339106 3080101

[42] Breyer JA. Diabetic nephropathy in insulin-dependent patients. Am J Kidney Dis (1992) 20:533–47. doi: 10.1016/S0272-6386(12)70215-9 1462980

[43] Ramsay RC. Progression of diabetic retinopathy after pancreas transplantation for insulin-dependent diabetes mellitus. N Engl J Med (1988) 318:208–14. doi: 10.1056/NEJM198801283180403 3275895

[44] Fajardo RJ. Is diabetic skeletal fragility associated with microvascular complications in bone? Curr Osteoporos Rep (2017) 15:1–8. doi: 10.1007/s11914-017-0341-8 28110469

[45] Oikawa A. Diabetes mellitus induces bone marrow microangiopathy. Arterioscler Thromb Vasc Biol (2010) 30:498–508. doi: 10.1161/ATVBAHA.109.200154 20042708 PMC3548136

[46] Stabley JN Prisby RD Behnke BJ Delp MD. Type 2 diabetes alters bone and marrow blood flow and vascular control mechanisms in the ZDF rat. J Endocrinol (2015) 225:47–58. doi: 10.1530/JOE-14-0514 25817711 PMC4379453

[47] Spinetti G. Global remodeling of the vascular stem cell niche in bone marrow of diabetic patients: implication of the microRNA-155/FOXO3a signaling pathway. Circ Res (2013) 112:510–22. doi: 10.1161/CIRCRESAHA.112.300598 PMC3616365 23250986

[48] Giannattasio C. Early impairment of large artery structure and function in type I diabetes mellitus. Diabetologia (1999) 42:987–94. doi: 10.1007/s001250051257 10491759

[49] Giannattasio C Failla M Grappiolo A Gamba PL Paleari F Mancia G. Progression of large artery structural and functional alterations in Type I diabetes. Diabetologia (2001) 44:203–8. doi: 10.1007/s001250051600 11270677

[50] Dong P Liu M Liu C. Exenatide inhibits the KCa3.1 channels of aortic vascular smooth muscle in diabetic rats. Acta Cardiol Sin (2017) 33:648–55. doi: 10.6515/ACS20170612B PMC5694930 29167619

[51] McCarthy I. The physiology of bone blood flow: a review. J Bone Joint Surg Am (2006) 88 Suppl:3, 4–9. doi: 10.2106/00004623-200611001-00002 17079361

[52] Bayliss WM. On the local reactions of the arterial wall to changes of internal pressure. J Physiol (1902) 28:220–31. doi: 10.1113/jphysiol.1902.sp000911 PMC1540533 16992618

[53] Mellander S. Functional aspects of myogenic vascular control. J Hypertens Suppl (1989) 7:S21–30. 2553897

[54] Kontos HA Wei EP Raper AJ Rosenblum WI Navari RM Patterson JL. Role of tissue hypoxia in local regulation of cerebral microcirculation. Am J Physiol (1978) 234:H582–591. doi: 10.1152/ajpheart.1978.234.5.H582 645924

[55] Proctor DN Parker BA. Vasodilation and vascular control in contracting muscle of the aging human. Microcirculation (2006) 13:315–27. doi: 10.1080/10739680600618967 16611597

[56] Walmsley D Wiles PG. Myogenic microvascular responses are impaired in long-duration type 1 diabetes. Diabetes Med (1990) 7:222–7. doi: 10.1111/j.1464-5491.1990.tb01374.x 2139393

[57] Newrick PG Cochrane T Betts RP Ward JD Boulton AJ. Reduced hyperaemic response under the diabetic neuropathic foot. Diabetes Med (1988) 5:570–3. doi: 10.1111/j.1464-5491.1988.tb01053.x 2974780

[58] Tooke JE Ostergren J Lins PE Fagrell B. Skin microvascular blood flow control in long duration diabetics with and without complications. Diabetes Res (1987) 5:189–92. 3311558

[59] Tecilazich F Feke GT Mazzantini S Sobrin L Lorenzi M. Defective myogenic response of retinal vessels is associated with accelerated onset of retinopathy in type 1 diabetic individuals. Invest Ophthalmol Vis Sci (2016) 57:1523–9. doi: 10.1167/iovs.15-18356 27035625

[60] Faris I Vagn Nielsen H Henriksen O Parving HH Lassen NA. Impaired autoregulation of blood flow in skeletal muscle and subcutaneous tissue in long-term Type 1 (insulin-dependent) diabetic patients with microangiopathy. Diabetologia (1983) 25:486–8. doi: 10.1007/BF00284456 6662278

[61] Nuutila P. Insulin resistance is localized to skeletal but not heart muscle in type 1 diabetes. Am J Physiol (1993) 264:E756–762. doi: 10.1152/ajpendo.1993.264.5.E756 8498497

[62] Baron AD Laakso M Brechtel G Edelman SV. Mechanism of insulin resistance in insulin-dependent diabetes mellitus: a major role for reduced skeletal muscle blood flow. J Clin Endocrinol Metab (1991) 73:637–43. doi: 10.1210/jcem-73-3-637 1874938

[63] Vallance P Collier J Moncada S. Effects of endothelium-derived nitric oxide on peripheral arteriolar tone in man. Lancet (1989) 2:997–1000. doi: 10.1016/S0140-6736(89)91013-1 2572793

[64] Palmer RM Ferrige AG Moncada S. Nitric oxide release accounts for the biological activity of endothelium-derived relaxing factor. Nature (1987) 327:524–6. doi: 10.1038/327524a0 3495737

[65] Ignarro LJ Buga GM Wood KS Byrns RE Chaudhuri G. Endothelium-derived relaxing factor produced and released from artery and vein is nitric oxide. Proc Natl Acad Sci USA (1987) 84:9265–9. doi: 10.1073/pnas.84.24.9265 PMC299734 2827174

[66] Yaqoob M. Relationship between markers of endothelial dysfunction, oxidant injury and tubular damage in patients with insulin-dependent diabetes mellitus. Clin Sci (Lond) (1993) 85:557–62. doi: 10.1042/cs0850557 8287643

[67] Myrup B Mathiesen ER Rønn B Deckert T. Endothelial function and serum lipids in the course of developing microalbuminuria in insulin-dependent diabetes mellitus. Diabetes Res (1994) 26:33–9. 7664535

[68] Mäkimattila S. Chronic hyperglycemia impairs endothelial function and insulin sensitivity *via* different mechanisms in insulin-dependent diabetes mellitus. Circulation (1996) 94:1276–82. doi: 10.1161/01.CIR.94.6.1276 8822980

[69] Huszka M. The association of reduced endothelium derived relaxing factor-NO production with endothelial damage and increased in *vivo* platelet activation in patients with diabetes mellitus. Thromb Res (1997) 86:173–80. doi: 10.1016/S0049-3848(97)00060-1 9175238

[70] Huvers FC. Endothelium-dependent vasodilatation, plasma markers of endothelial function, and adrenergic vasoconstrictor responses in type 1 diabetes under near-normoglycemic conditions. Diabetes (1999) 48:1300–7. doi: 10.2337/diabetes.48.6.1300 10342820

[71] Järvisalo MJ. Endothelial dysfunction and increased arterial intima-media thickness in children with type 1 diabetes. Circulation (2004) 109:1750–5. doi: 10.1161/01.CIR.0000124725.46165.2C 15023875

[72] Mahmud FH Earing MG Lee RA Lteif AN Driscoll DJ Lerman A. Altered endothelial function in asymptomatic male adolescents with type 1 diabetes. Congenit Heart Dis (2006) 1:98–103. doi: 10.1111/j.1747-0803.2006.00015.x 18377552

[73] Wink DA. Mechanisms of the antioxidant effects of nitric oxide. Antioxid Redox Signal (2001) 3:203–13. doi: 10.1089/152308601300185179 11396476

[74] de A. do Nascimento AMM Sequeira IJ Vasconcelos DF Gandolfi L Pratesi R de M. Nóbrega YK. Endothelial dysfunction in children with type 1 diabetes mellitus. Arch Endocrinol Metab (2017) 61:476–83. doi: 10.1590/2359-3997000000271 PMC10522258 28658349

[75] Cé GV. Endothelial dysfunction is related to poor glycemic control in adolescents with type 1 diabetes under 5 years of disease: evidence of metabolic memory. J Clin Endocrinol Metab (2011) 96:1493–9. doi: 10.1210/jc.2010-2363 21346068

[76] Babar GS. Impaired endothelial function in preadolescent children with type 1 diabetes. Diabetes Care (2011) 34:681–5. doi: 10.2337/dc10-2134 PMC3041207 21289230

[77] Lespagnol E. Early endothelial dysfunction in type 1 diabetes is accompanied by an impairment of vascular smooth muscle function: A meta-analysis. Front Endocrinol (Lausanne) (2020) 11:203. doi: 10.3389/fendo.2020.00203 32362871 PMC7180178

[78] Clarkson P. Impaired vascular reactivity in insulin-dependent diabetes mellitus is related to disease duration and low density lipoprotein cholesterol levels. J Am Coll Cardiol (1996) 28:573–9. doi: 10.1016/0735-1097(96)82380-1 8772741

[79] Jin SM. Endothelial dysfunction and microvascular complications in type 1 diabetes mellitus. J Korean Med Sci (2008) 23:77–82. doi: 10.3346/jkms.2008.23.1.77 18303203 PMC2526502

[80] Elliott TG Cockcroft JR Groop PH Viberti GC Ritter JM. Inhibition of nitric oxide synthesis in forearm vasculature of insulin-dependent diabetic patients: blunted vasoconstriction in patients with microalbuminuria. Clin Sci (Lond) (1993) 85:687–93. doi: 10.1042/cs0850687 8287660

[81] Prisby RD. Aging reduces skeletal blood flow, endothelium-dependent vasodilation, and NO bioavailability in rats. J Bone Miner Res (2007) 22:1280–8. doi: 10.1359/jbmr.070415 17451371

[82] Azuma H. INTRAOSSEOUS PRESSURE AS A MEASURE OF HEMODYNAMIC CHANGES IN BONE MARROW. Angiology (1964) 15:396–406. doi: 10.1177/000331976401500903 14210346

[83] Gross PM Heistad DD Marcus ML. Neurohumoral regulation of blood flow to bones and marrow. Am J Physiol (1979) 237:H440–448. doi: 10.1152/ajpheart.1979.237.4.H440 495729

[84] Shaw NE. Observations on the intramedullary blood-flow and marrow-pressure in bone. Clin Sci (1963) 24:311–8. 13976967

[85] Stein AH Morgan HC Porras RF. The effect of pressor and depressor drugs on intramedullary bone-marrow pressure. J Bone Joint Surg Am (1958) 40-A:1103–10. doi: 10.2106/00004623-195840050-00012 13587578

[86] Dean MT Wood MB Vanhoutte PM. Antagonist drugs and bone vascular smooth muscle. J Orthop Res (1992) 10:104–11. doi: 10.1002/jor.1100100113 1727930

[87] Driessens M Vanhoutte PM. Vascular reactivity of the isolated tibia of the dog. Am J Physiol (1979) 236:H904–908. doi: 10.1152/ajpheart.1979.236.6.H904 443457

[88] Ye Z Wood MB Vanhoutte PM. Alpha-adrenergic receptor responsiveness in vascular smooth muscle of canine bone. Clin Orthop Relat Res (1993) 287:286–91. doi: 10.1097/00003086-199302000-00044 8095442

[89] Dominguez JM Prisby RD Muller-Delp JM Allen MR Delp MD. Increased nitric oxide-mediated vasodilation of bone resistance arteries is associated with increased trabecular bone volume after endurance training in rats. Bone (2010) 46:813–9. doi: 10.1016/j.bone.2009.10.029 PMC2823927 19892040

[90] Duncker DJ Stubenitsky R Tonino PA Verdouw PD. Nitric oxide contributes to the regulation of vasomotor tone but does not modulate O(2)-consumption in exercising swine. Cardiovasc Res (2000) 47:738–48. doi: 10.1016/s0008-6363(00)00143-7 10974222

[91] Iversen PO Nicolaysen G Benestad HB. Endogenous nitric oxide causes vasodilation in rat bone marrow, bone, and spleen during accelerated hematopoiesis. Exp Hematol (1994) 22:1297–302. 7957715

[92] Heinonen I Boushel R Hellsten Y Kalliokoski K. Regulation of bone blood flow in humans: The role of nitric oxide, prostaglandins, and adenosine. Scand J Med Sci Sports (2018) 28:1552–8. doi: 10.1111/sms.13064 29377406

[93] Colleran PN Wilkerson MK Bloomfield SA Suva LJ Turner RT Delp MD. Alterations in skeletal perfusion with simulated microgravity: a possible mechanism for bone remodeling. J Appl Physiol (1985) (2000) 89:1046–54. doi: 10.1152/jappl.2000.89.3.1046 10956349

[94] Vogt MT Cauley JA Kuller LH Nevitt MC. Bone mineral density and blood flow to the lower extremities: the study of osteoporotic fractures. J Bone Miner Res (1997) 12:283–9. doi: 10.1359/jbmr.1997.12.2.283 9041062

[95] Burkhardt R. Changes in trabecular bone, hematopoiesis and bone marrow vessels in aplastic anemia, primary osteoporosis, and old age: a comparative histomorphometric study. Bone (1987) 8:157–64. doi: 10.1016/8756-3282(87)90015-9 3606907

[96] Lakatta EG Levy D. Arterial and cardiac aging: major shareholders in cardiovascular disease enterprises: Part I: aging arteries: a ‘set up’ for vascular disease. Circulation (2003) 107:139–46. doi: 10.1161/01.CIR.0000048892.83521.58 12515756

[97] Atkinson J. Arterial calcification. Mechanisms, consequences and animal models. Pathol Biol (Paris) (1999) 47:677–84. 10522258

[98] Snell-Bergeon JK Budoff MJ Hokanson JE. Vascular calcification in diabetes: mechanisms and implications. Curr Diabetes Rep (2013) 13:391–402. doi: 10.1007/s11892-013-0379-7 23526400

[99] Schurgin S Rich S Mazzone T. Increased prevalence of significant coronary artery calcification in patients with diabetes. Diabetes Care (2001) 24:335–8. doi: 10.2337/diacare.24.2.335 11213888

[100] Kauffenstein G. Disseminated arterial calcification and enhanced myogenic response are associated with abcc6 deficiency in a mouse model of pseudoxanthoma elasticum. Arterioscler Thromb Vasc Biol (2014) 34:1045–56. doi: 10.1161/ATVBAHA.113.302943 PMC4341999 24675664

[101] Majumdar U. Nitric oxide prevents aortic valve calcification by S-nitrosylation of USP9X to activate NOTCH signaling. Sci Adv (2021) 7:eabe3706. doi: 10.1126/sciadv.abe3706 33547080 PMC7864581

[102] Kanno Y Into T Lowenstein CJ Matsushita K. Nitric oxide regulates vascular calcification by interfering with TGF- signalling. Cardiovasc Res (2008) 77:221–30. doi: 10.1093/cvr/cvm049 18006450

[103] Park J-H Iemitsu M Maeda S Kitajima A Nosaka T Omi N. Voluntary running exercise attenuates the progression of endothelial dysfunction and arterial calcification in ovariectomized rats. Acta Physiol (Oxf) (2008) 193:47–55. doi: 10.1111/j.1748-1716.2007.01799.x 18005246

[104] Oe Y. Lack of endothelial nitric oxide synthase accelerates ectopic calcification in uremic mice fed an adenine and high phosphorus diet. Am J Pathol (2021) 191:283–93. doi: 10.1016/j.ajpath.2020.10.012 33159888

[105] Tesauro M. Arterial ageing: from endothelial dysfunction to vascular calcification. J Intern Med (2017) 281:471–82. doi: 10.1111/joim.12605 28345303

[106] Khandkar C Vaidya K Karimi Galougahi K Patel S. Low bone mineral density and coronary artery disease: A systematic review and meta-analysis. Int J Cardiol Heart Vasc (2021) 37:100891. doi: 10.1016/j.ijcha.2021.100891 34746361 PMC8554269

[107] Zhang Y Feng B. Systematic review and meta-analysis for the association of bone mineral density and osteoporosis/osteopenia with vascular calcification in women. Int J Rheum Dis (2017) 20:154–60. doi: 10.1111/1756-185X.12842 27153243

[108] Chan JJ Cupples LA Kiel DP O’Donnell CJ Hoffmann U Samelson EJ. QCT volumetric bone mineral density and vascular and valvular calcification: the framingham study. J Bone Miner Res (2015) 30:1767–74. doi: 10.1002/jbmr.2530 PMC4809363 25871790

[109] Burghardt AJ Buie HR Laib A Majumdar S Boyd SK. Reproducibility of direct quantitative measures of cortical bone microarchitecture of the distal radius and tibia by HR-pQCT. Bone (2010) 47:519–28. doi: 10.1016/j.bone.2010.05.034 PMC2926164 20561906

[110] Boutroy S Bouxsein ML Munoz F Delmas PD. *In vivo* assessment of trabecular bone microarchitecture by high-resolution peripheral quantitative computed tomography. J Clin Endocrinol Metab (2005) 90:6508–15. doi: 10.1210/jc.2005-1258 16189253

[111] Patsch JM. Quantification of lower leg arterial calcifications by high-resolution peripheral quantitative computed tomography. Bone (2014) 58:42–7. doi: 10.1016/j.bone.2013.08.006 PMC4042679 23954758

[112] Cejka D Weber M Diarra D Reiter T Kainberger F Haas M. Inverse association between bone microarchitecture assessed by HR-pQCT and coronary artery calcification in patients with end-stage renal disease. Bone (2014) 64:33–8. doi: 10.1016/j.bone.2014.03.048 24709688

[113] Paccou J. Lower leg arterial calcification assessed by high-resolution peripheral quantitative computed tomography is associated with bone microstructure abnormalities in women. Osteoporos Int (2016) 27:3279–87. doi: 10.1007/s00198-016-3660-1 PMC5040512 27325126

[114] Salam S Gallagher O Gossiel F Paggiosi M Eastell R Khwaja A. Vascular calcification relationship to vascular biomarkers and bone metabolism in advanced chronic kidney disease. Bone (2021) 143:115699. doi: 10.1016/j.bone.2020.115699 33091638

